# Evidence of Omics, Immune Infiltration, and Pharmacogenomics for BATF in a Pan-Cancer Cohort

**DOI:** 10.3389/fmolb.2022.844721

**Published:** 2022-04-29

**Authors:** Chenguang Jia, Yihui Ma, Mengyang Wang, Wen Liu, Feng Tang, Jincao Chen

**Affiliations:** Department of Neurosurgery, Zhongnan Hospital of Wuhan University, Wuhan, China

**Keywords:** BATF, immune-cell infiltration, immunotherapy, pan-cancer, biomarker

## Abstract

**Background:** Cytotoxic CD8+ T-cell exhaustion is the major barrier for immunotherapy in tumors. Recent studies have reported that the basic leucine zipper activating transcription factor–like transcription factor (BATF) is responsible for countering cytotoxic CD8+ T-cell exhaustion. Nevertheless, the expression and roles of BATF in tumors have been poorly explored.

**Methods:** In the present study, we conducted a multi-omics analysis, including gene expression, methylation status, DNA alterations, pharmacogenomics, and survival status based on data from the Cancer Genome Atlas (TCGA) database to discern expression patterns and prognostic roles of BATF in tumors. We also explored potential roles of BATF in a pan-cancer cohort by performing immune infiltration, Gene Ontology (GO) enrichment, and the Kyoto Encyclopedia of Genes and Genomes (KEGG) analysis. *In vitro* assay was also performed to explore roles of BATF in tumor cells.

**Results:** We found that BATF was aberrantly upregulated in 27 types of tumors with respect to the corresponding normal tissues. Abnormal BATF expression in tumors predicted survival times of patients in a tissue-dependent manner. The results of GO, immune infiltration, and KEGG analysis revealed that increased BATF expression in tumors participated in modulating immune cell infiltration *via* immune-related pathways. BATF expression could also predict immunotherapeutic and chemotherapy responses in cancers. Moreover, knockdown of BATF suppresses tumor cell viability.

**Conclusion:** Our present study reports the vital roles of BATF in tumors and provides a theoretical basis for targeting BATF therapy.

## Introduction

CD8+ T cells represent a vital immune cell populations associated with immune responses in the majority of cancers (Chen and Mellman, 2013). Upon encountering neoantigens presented by tumor cells, CD8+ T cells are activated and then differentiate into cytotoxic CD8+ T cells (CTLs) to clear cancer cells in the early stage. The prolonged dominant antigenic stimulation, in turn, leads to a dysfunctional hyporesponsiveness (or “exhaustion”) of CTLs (Jiang et al., 2015; Schietinger and Greenberg, 2014). The exhausted CTLs increasingly fail to effectively destroy tumor cells, resulting in tumor immune evasion (Jiang et al., 2020). Recently, two published studies reported that basic leucine zipper activating transcription factor (ATF)–like transcription factor (BATF) is crucial for regulating the progenitor to CTL transition and countering CD8+ T-cell exhaustion. In addition, BATF-transduced CD8+ T-cells containing a chimeric antigen receptor (CAR) exhibited enhanced tumor rejection and expansion of tumor-infiltrating CAR T-cells (Chen et al., 2021; Seo et al., 2021). BATF-transduced CAR T-cells may improve immunotherapy responses in patients with tumors in the clinic.

BATF protein family including BATF, BATF2, and BATF3, is one subfamily of the activator protein 1 (AP-1)/ATF superfamily of transcription factors. Among the three BATFs, BATF is the only negative regulator of AP-1/ATF transcriptional events (Murphy et al., 2013). In tumors, abnormal BATF expression was found in anaplastic large-cell lymphoma and non-small-cell lung (Schleussner et al., 2018; Feng et al., 2020). Knockdown or overexpression of BATF also participates in regulating cell progression, apoptosis, proliferation and invasion in several tumors (Punkenburg et al., 2016; Schleussner et al., 2018; Feng et al., 2020; Zhang et al., 2021). However, the expression and potential roles (especially immune cell infiltration) of BATF in the majority of cancers remains to be illustrated. A pan-cancer omics analysis to explore potential roles of BATF in tumors may provide a novel strategy for tumor immunotherapy.

In this study, we employed a multi-omics analysis of BATF in carcinogenesis, including gene expression, methylation status, DNA alterations, immune infiltration, pharmacogenomics, and survival status based on data from the Cancer Genome Atlas (TCGA) database. Such a pan-cancer analysis provides evidence to uncover the distinct roles of BATF in the immune microenvironment, which may be meaningful for the following functional experiments.

## Materials and Methods

### Gene Expression Analysis

We first used the online TIMER2 (http://timer.cistrome.org/) webserver tool to explore the differential BATF expression between 33 TCGA tumor types and corresponding adjacent normal tissues (Li et al., 2020). Next, we validated the expression profile of BATF between the tumor tissues and corresponding normal tissues based on tumor data from the TCGA database and Genotype-tissue Expression (GTEx) database, respectively. The “Pathological Stage Plot” module of GEPIA2 tool (http://gepia2.cancer-pku.cn/#about) was used to obtain plots of the BATF expression in various pathological stages of TCGA tumors (Tang et al., 2019). When *p* < 0.05, it was considered to be statistically significant.

### Survival Prognosis Analysis

The “Gene_Outcome” module of TIMER2 was applied to assess the clinical relevance of BATF expression across 33 type tumors. A Cox proportional hazards model was conducted to explore the outcome significance of BATF expression and a heatmap was drawn to show the normalized coefficient of BATF in the Cox model. According to median expression levels of BATF, each type of tumors was divided into high-expression and low-expression groups. After that, survival analysis of patients with tumors was conducted. A Kaplan–Meier plotter curve of BATF was created to evaluate the relationship between overall survival and BATF expression in TCGA tumors (Li et al., 2020). When *p* < 0.05, it was considered to be statistically significant.

### Methylation Analysis

We investigated the methylation of BATF in the 33 type tumors and corresponding adjacent normal tissues using the UALCAN portal (http://ualcan.path.uab.edu/). UALCAN is a comprehensive, user-friendly, and interactive web resource for analyzing cancer omics data (Chandrashekar et al., 2017). We applied the University of California, Santa Cruz’s Xena (https://xenabrowser.net/heatmap/) tool to analyze the relationship between survival time and BATF methylation levels in the corresponding tumors. The Xena is a platform, which provides huge amounts of processed cancer omics data from large cancer research projects (e.g., TCGA, CCLE, and PCAWG) (Wang et al., 2021). When *p* < 0.05, it was considered to be statistically significant.

### Genetic-Alteration Analysis

We used the cBioPortal portal (https://www.cbioportal.org/) to investigate genetic alterations of the BATF gene in the pan-cancer cohort. We identified genetic alterations, such as structural variants, mutation type, and copy number alterations, in the 33 TCGA tumor types *via* the “Cancer Types Summary” module. We also applied the “Comparison” module to gain insights into the effects of BATF genetic alterations on the overall, progression-free, disease-specific, and disease-free survival rates. When *p* < 0.05, it was considered to be statistically significant.

### Gene Ontology Enrichment and Kyoto Encyclopedia of Genes and Genomes Analysis

We applied the “Similar Gene Detection” module of the GEPIA2 to obtain the top-100 BATF-correlated targeting genes across all the TCGA tumor samples (Tang et al., 2019). Using these 100 correlated genes, we performed GO enrichment and KEGG analysis using the R software program (R Foundation for Statistical Computing, Vienna, Austria). A *p*-value < 0.05 was considered to be statistically significant.

### Immune-Infiltration Analysis

Data on the 33 tumor patients in the TCGA database, tumor RNA-seq data (TCGA) and BATF data can be downloaded from the Genomic Data Commons data (https://portal.gdc.cancer.gov/) portal website. Each tumor had messenger RNA expression data available from matched normal tissue samples. For reliable immune score evaluation, we used immuneeconv, an R software package that integrates six newer algorithms, including TIMER, xCell, MCP-counter, CIBERSORT, EPIC, and quanTIseq (Li et al., 2016; Aran et al., 2017; Sturm et al., 2019). When *p* < 0.05 and R-values between −1 and one were obtained, viz. the Spearman’s rank correlation test, and were considered to be statistically significant.

### Drug-Sensitivity Analysis

We applied the “Drug” module of the Gene Set Cancer Analysis (GSCA) to obtain the correlation between BATF expression and drug sensitivity (IC_50_) of updated new drugs. GSCA integrates more than 10,000 multi-dimensional genomic data across 33 cancer types from TCGA and more than 750 small molecule drugs from Genomics of Drug Sensitivity in Cancer (GDSC) and Cancer Therapeutics Response Portal (CTRP) (Liu et al., 2018).

### Cell Culture and Transfection

Glioma U251 and gastric cancer SGC7901 cells were purchased from ATCC. Cells were cultured with DMEM medium (Gibco) containing 10% fetal bovine serum (Gibco) and 1% penicillin and streptomycin (Sigma). These cells were cultivated in cell incubator with 5% CO_2_ at 37°C. 2000 cells/well were seeded in 96-well plates and cultured for 24 h before transfection. After that, BATF siRNA were transfected with Lip3000 (Thermo Fisher Scientific) according to the manufacturer’s instructions.

### RT-qPCR

Trizol reagent (Invitrogen) was applied to extract total RNA from tumor cells. Reverse transcription and Real-time PCR was conducted by using a PrimeScript RT reagent kit (Takara) and SYBR Premix Ex Taq II (Takara), respectively. The primers of BATF was were synthesized from Sangon Biotech (Forward: 5′-CCC​TGG​CAA​ACA​GGA​CTC​AT-3′; Reverse: 5′- GAT​CTC​CTT​GCG​TAG​AGC​CG-3′). The relative expression levels were normalized by using the 2^−ΔΔCt^ method.

### CCK-8 Assay

48 h after transfection, cell culture medium were replaced with fresh medium and cultured for indicated times. 10 μl of CCK-8 solution was added to each well and incubated for 1 h at the same incubator conditions. After that, the absorbance at 450 nm was detected with a micro-plate analyzer.

### Statistical Analysis

SPSS software (ver. 21.0; SPSS, Chicago, IL) were applied for statistical analyses and the significance was determined by one-way ANOVA or t-test. A *p*-value < 0.05 was considered to be statistically significant.

## Results

### Expression Profiles of BATF in the Pan-Cancer Cohort

**FIGURE 1:**
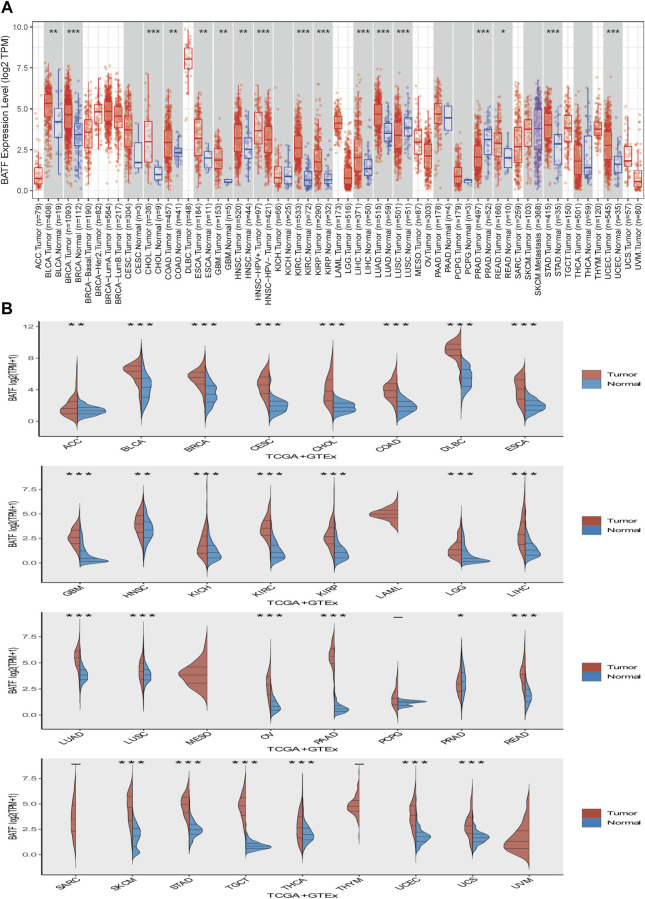
Expression of BATF in pan-cancer. **(A)** BATF expression levels in tumor tissues and corresponding adjacent tissues. **(B)** BATF expression levels in tumor tissues and corresponding normal tissues.

**FIGURE 2:**
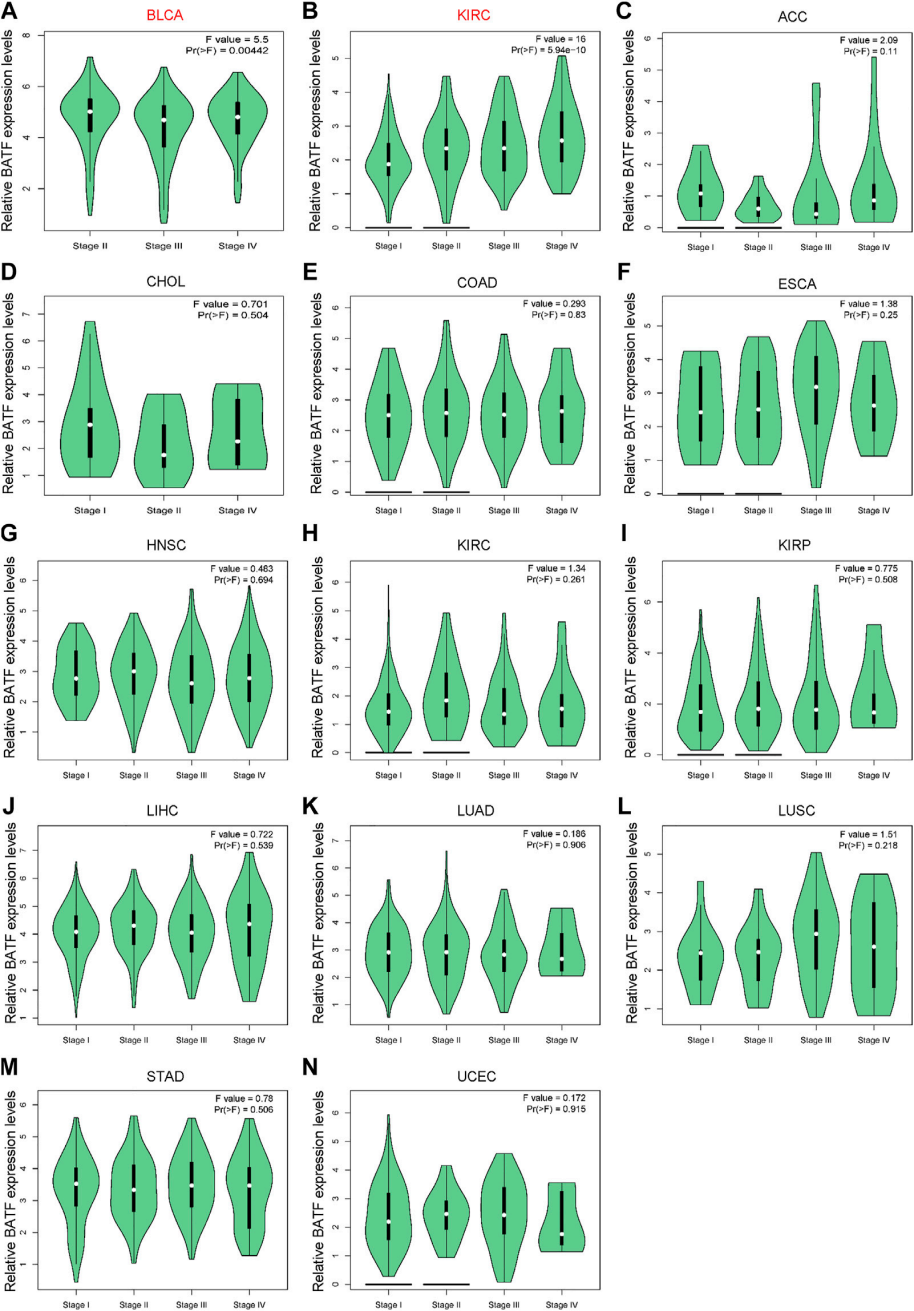
Correlation between aberrant BATF expression and clinicopathological stages in tumors. Red fonts indicate significant differences among these groups, while black fonts indicate no statistically significant differences.

First, we explored expression levels of BATF in normal tissues and single cells. As shown in Supplementary Figure S1A, BATF expression in areas of the central nervous system, such as the cerebral cortex and cerebellum, was low, while it was more highly expressed in organs of the immune system, like the spleen. In most cells, such as neuronal cells and glial cells, BATF is retained at very low expression levels. In contrast, immune cells—for instance, B-cells, T-cells, and granulocytes—present high expression levels, which may explain the roles of BATF in normal immune cells (Supplementary Figure S1B). Next, we compared the expression levels of BATF in 33 tumor types and adjacent normal tissues. As shown in Figure 1A, we found that BATF was upregulated in bladder urothelial carcinoma (BLCA), lung adenocarcinoma (LUAD), stomach adenocarcinoma (STAD), esophageal carcinoma (ESCA), breast invasive carcinoma (BRCA), cholangiocarcinoma, kidney renal papillary cell carcinoma (KIRP), glioblastoma multiforme, prostate adenocarcinoma (PRAD), kidney renal clear cell carcinoma (KIRC), colon adenocarcinoma (COAD), head and neck squamous cell carcinoma (HNSC), lung squamous cell carcinoma (LUSC), liver hepatocellular carcinoma (LIHC), rectum adenocarcinoma (READ), and uterine corpus endometrial carcinoma (UCEC). Compared with the corresponding normal tissues, BATF was abnormally expressed in 27 TCGA tumor types, including cervical squamous cell carcinoma and endocervical adenocarcinoma (CESC), ower-grade glioma (LGG), adrenocortical carcinoma (ACC), thymoma, UCEC, l pancreatic adenocarcinoma (PAAD), cutaneous melanoma (SKCM), uterine carcinosarcoma (UCS), lymphoid neoplasm diffuse large B-cell lymphoma (DLBC), kidney chromophobe, ovarian serous cystadenocarcinoma (OV), STAD, testicular germ cell tumors (TGCT), and thyroid carcinoma (THCA). We also explored mRNA expression levels of BATF in cancer cell lines of different tumors *via* the CCLE database (https://portals.broadinstitute.org/ccle/home). The CCLE database is a public database that contains expression data of more than 30 types of tumors (including glioma and gastric cancer), 1457 tumor cell lines. Similarly, BATF was also highly expressed in several tumor cells, and BATF protein was dominantly distributed in the nucleus of these cells (Supplementary Figure S2). Interestingly, we found that BATF expression was only correlated with BLCA and KIRC clinicopathological stages; in other tumors, BATF expression levels showed no correlation with tumor stages (Figure 2). These results indicated that BATF was indeed upregulated in most tumors, and the forced BATF expression was unlikely associated with tumor progression, except for BLCA and KIRC.

### Prognostic Value of BATF in Cancers

**FIGURE 3:**
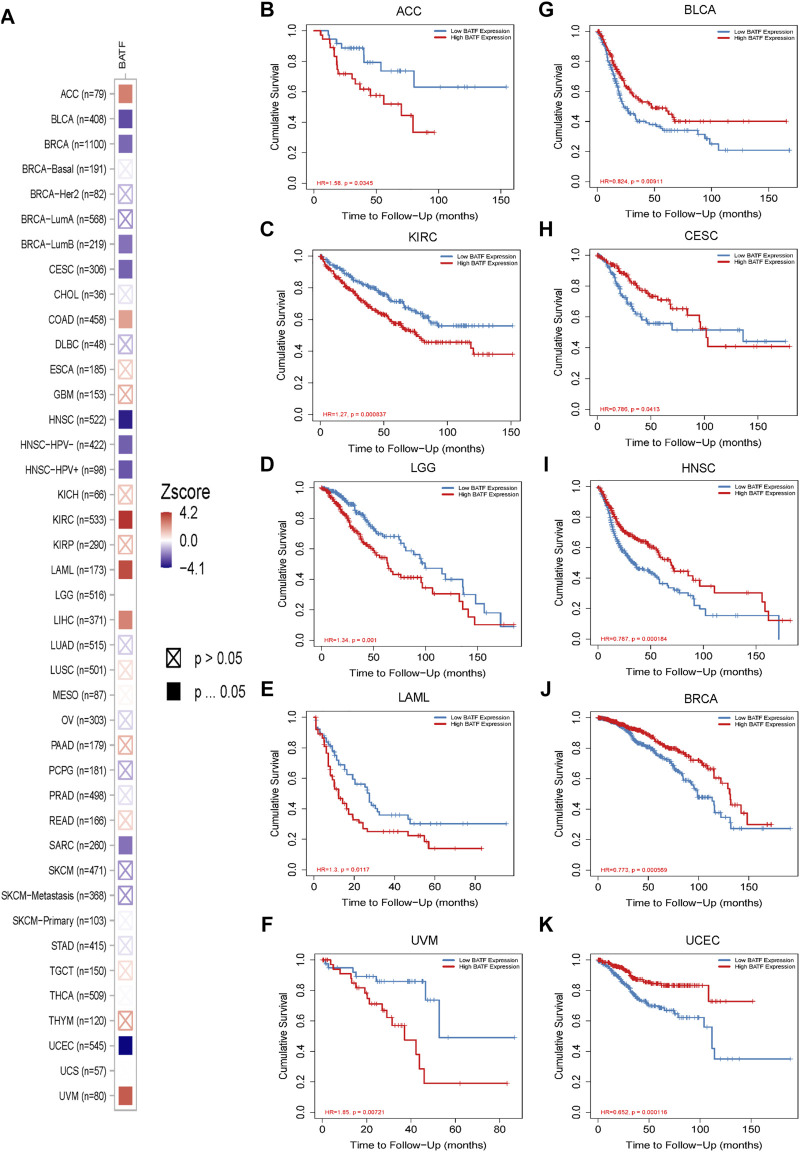
Prognostic roles of BATF expression in tumors. **(A)** Cox regression analysis of BATF expression in pan-cancer. **(B–K)** Kaplan-Meier Plotter analysis of BATF expression in tumors.

The prognostic role of BATF in tumors is presented in Figure 3. The Cox regression result showed that BATF was a high-risk indicator in LAML, ACC, LGG, COAD, LIHC, KIRC, and UVM. On the contrary, upregulated BATF was a favorable factor in HNSC, CESC, BRCA, SARC, BLCA, and UCEC (Figure 3A and Table 1). We next divided the tumor species into high- and low-expression groups according to median expression levels of BATF to evaluate the correlation between BATF expression and survival times of patients with tumors. We found that patients with KIRC, LGG, LAML, ACC, and UVM and higher BATF expression levels had poorer overall survival. In contrast, a higher expression of BATF was associated with a significantly better prognosis in HNSC, BLCA, BRCA, CESC, and UCEC. The results of Kaplan–Meier plotter analysis were in line with the results of Cox regression analysis (Figures 3B–K). These results indicated that the prognostic role of BATF in tumors is tissue-dependent.

**TABLE 1:** Cox regression analysis of BATF in cancers.

Tumor	Hazard ratio	Z Score	*p* value
ACC	1.475 (1.104–1.971)	2.629	0.009
BLCA	0.846 (0.755–0.948)	−2.877	0.004
BRCA	0.861 (0.76–0.974)	−2.373	0.018
BRCA-Basal	0.964 (0.727–1.279)	−0.254	0.799
BRCA-Her2	0.705 (0.381–1.305)	−1.113	0.266
BRCA-LumA	0.83 (0.671–1.028)	−1.707	0.088
BRCA-LumB	0.722 (0.54–0.965)	−2.201	0.028
CESC	0.815 (0.693–0.959)	−2.462	0.014
CHOL	0.938 (0.681–1.292)	−0.39	0.697
COAD	1.18 (1.001–1.393)	1.967	0.049
DLBC	0.733 (0.418–1.286)	−1.082	0.279
ESCA	1.089 (0.925–1.281)	1.026	0.305
GBM	1.213 (0.963–1.529)	1.64	0.101
HNSC	0.803 (0.716–0.9)	−3.75	0.0001
HNSC-HPV+	0.66 (0.489–0.892)	−2.703	0.007
HNSC-HPV-	0.849 (0.747–0.964)	−2.516	0.012
KICH	1.429 (0.799–2.555)	1.202	0.229
KIRC	1.336 (1.162–1.536)	4.061	0.0001
KIRP	1.202 (0.947–1.525)	1.51	0.131
LAML	1.894 (1.346–2.665)	3.667	0.0002
LGG	1.541 (1.261–1.884)	4.226	0.0001
LIHC	1.17 (1.038–1.318)	2.57	0.01
LUAD	0.943 (0.814–1.093)	−0.777	0.437
LUSC	1.038 (0.919–1.172)	0.602	0.547
MESO	1.02 (0.823–1.264)	0.179	0.858
OV	0.947 (0.807–1.111)	−0.669	0.503
PAAD	1.139 (0.969–1.339)	1.582	0.114
PCPG	0.886 (0.076–1.696)	−1.294	0.195
PRAD	0.851 (0.422–1.718)	−0.449	0.653
READ	1.16 (0.833–1.616)	0.879	0.379
SARC	0.861 (0.754–0.984)	−2.203	0.028
SKCM	0.927 (0.849–1.012)	−1.697	0.09
SKCM-Primary	0.972 (0.714–1.323)	−0.182	0.855
SKCM-Metastasis	0.922 (0.841–1.012)	−1.704	0.088
STAD	0.966 (0.835–1.119)	−0.457	0.648
TGCT	1.372 (0.546–3.445)	0.673	0.501
THCA	0.979 (0.638–1.504)	−0.096	0.924
THYM	1.982 (0.988–3.976)	1.926	0.054
UCEC	0.678 (0.562–0.817)	−4.079	0.0001
UCS	1.004 (0.725–1.391)	0.024	0.981
UVM	1.688 (1.244–2.291)	3.364	0.001

### DNA Methylation Analysis of BATF in Cancers

**FIGURE 4:**
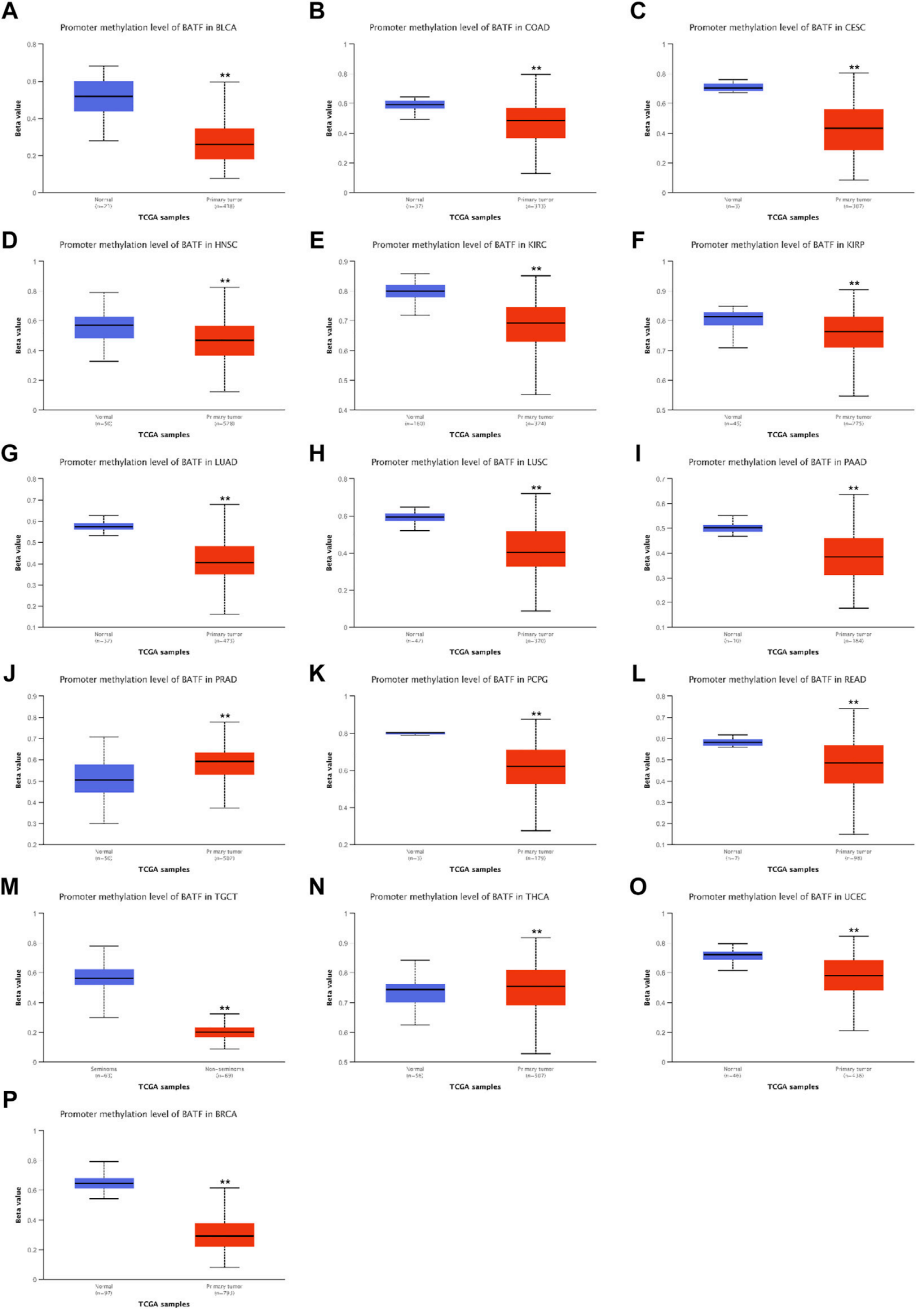
Promoter methylation levels of BATF in pan-cancer. **p < 0.01 compared with corresponding normal tissues.

DNA methylation is the most common way to regulate gene transcription, and we speculated that the dysregulated expression of BATF may be modulated in this manner. As shown in Figure 4, patients with BLCA, READ, LUAD, CESC, KIRC, PRAD, LUSC, PCPG, KIRP, TGCT, COAD, THCA, HNSC, and UCEC demonstrated lower methylation levels with respect to corresponding normal tissues, which may explain the higher BATF expression levels in these tumors. Notice that, although BATF was upregulated in PRAD samples, methylation levels of PRAD are higher than those in normal tissues (Figure 4J). Apart from DNA methylation, post-transcriptional modification like N6-methyladenosine (m6A) is a vital way to affect gene expression (Jiang et al., 2021). Abnormal expression of BATF in PRAD may be controlled in m6A-dependent manner. In ACC, BLCA, BRCA, CESC, HNSC, and UCEC, patients with higher levels of methylation of the *BATF* gene had shorter survival times. In contrast, lower methylation levels of the *BATF* gene expression predicted poorer overall survival in LGG and UVM (Supplementary Figure S3). These results suggest that increased BATF expression in tumors is probably due to the low methylation of its promoter in cancers other than PRAD. In addition, methylation levels predicting overall survival in tumor patients are also tissue-dependent.

### Genetic Alteration Analysis of BATF in Cancers

**FIGURE 5:**
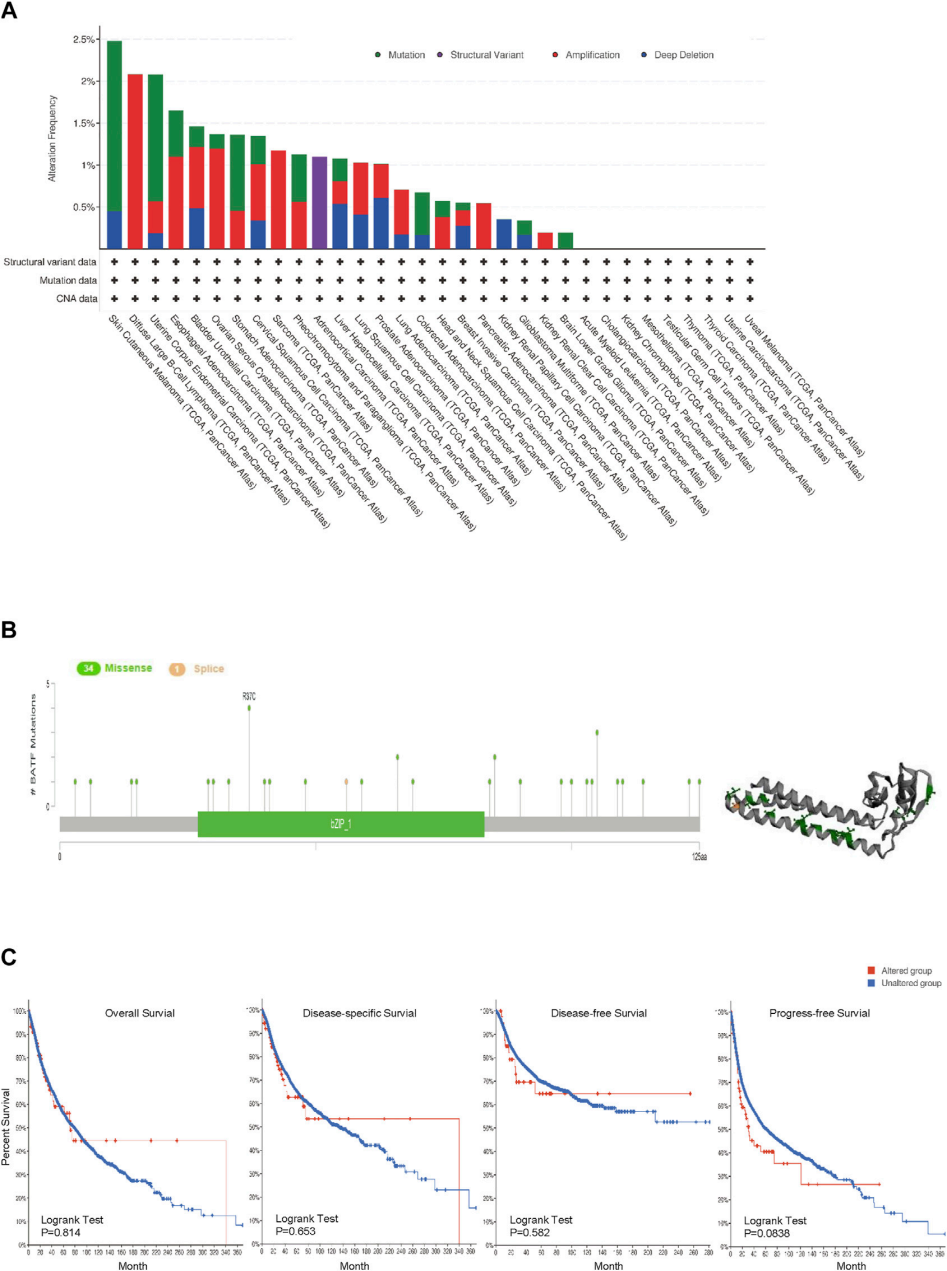
Genetic alterations in BATF in cancers. **(A,B)** Alteration frequency and mutation types of BATF in tumors. **(C)** OS, DSS, DFS, and PFS in tumors with BATF genetic alterations.

Gene alteration plays crucial roles in tumor development and progression. Hence, we investigated genetic alterations of BATF in a pan-cancer cohort containing 10,969 tumor samples. The top three genetic alterations with rates over 2% were SKCM, DLBL, and UCEC, respectively. Among the types of genetic alteration, mutation, amplification, and deep deletion account for almost all types in the majority of tumors; structural variations were only found in ACC (Figure 5A). There were 34 missense and one splice mutations, and R37c was the most common mutated site (Figure 5B). We found that there was no difference in overall survival (OS) rates between BATF-altered and -unaltered groups across different cancers (Figure 5C). It seems that genetic alterations of BATF play a limited role in carcinomas.

### BATF Expression was Related to Immune Infiltration

**FIGURE 6:**
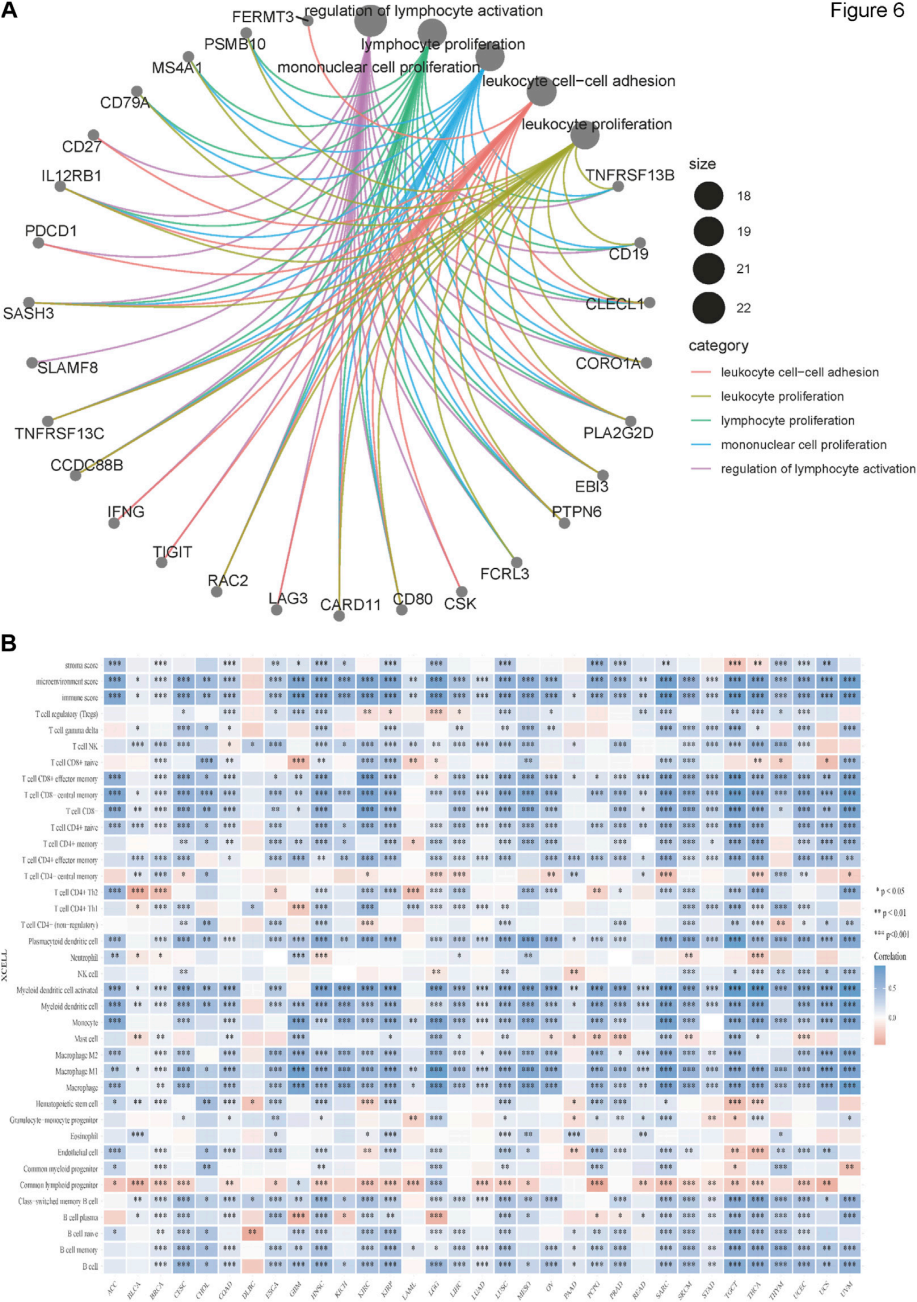
Immune infiltration of BATF in pan-cancer. **(A)** GO enrichment analysis of BATF. **(B)** Correlation between BATF expression and immune-cell infiltration.

We obtained the top 100 genes similar to BATF across 33 tumor types from the GEPIA2 “Similar genes” module and performed GO enrichment analysis to gain insights into the roles of BATF in tumors. As shown in Figure 6A, BATF expression was mainly involved in regulating leukocyte cell–cell adhesion, leukocyte proliferation, lymphocyte proliferation, mononuclear cell proliferation, and lymphocyte activation. To validate this result, we performed an immune-cell infiltration analysis. As shown in Figure 6B, there is a positive correlation between BATF expression and infiltration levels of most immune cells. However, BATF expression was negatively correlated with common lymphoid progenitor infiltration in most tumors. In DLBC, BATF expression was only correlated with several immune cell types, such as natural killer T-cells, CD4+ Th1 cells, hematopoietic stem cells, class-switched memory B-cells, and naive B-cells. These results demonstrate that BATF is involved in modulating immune infiltration in tumors.

### BATF Participates in Regulating Immune-Related Pathways

**FIGURE 7:**
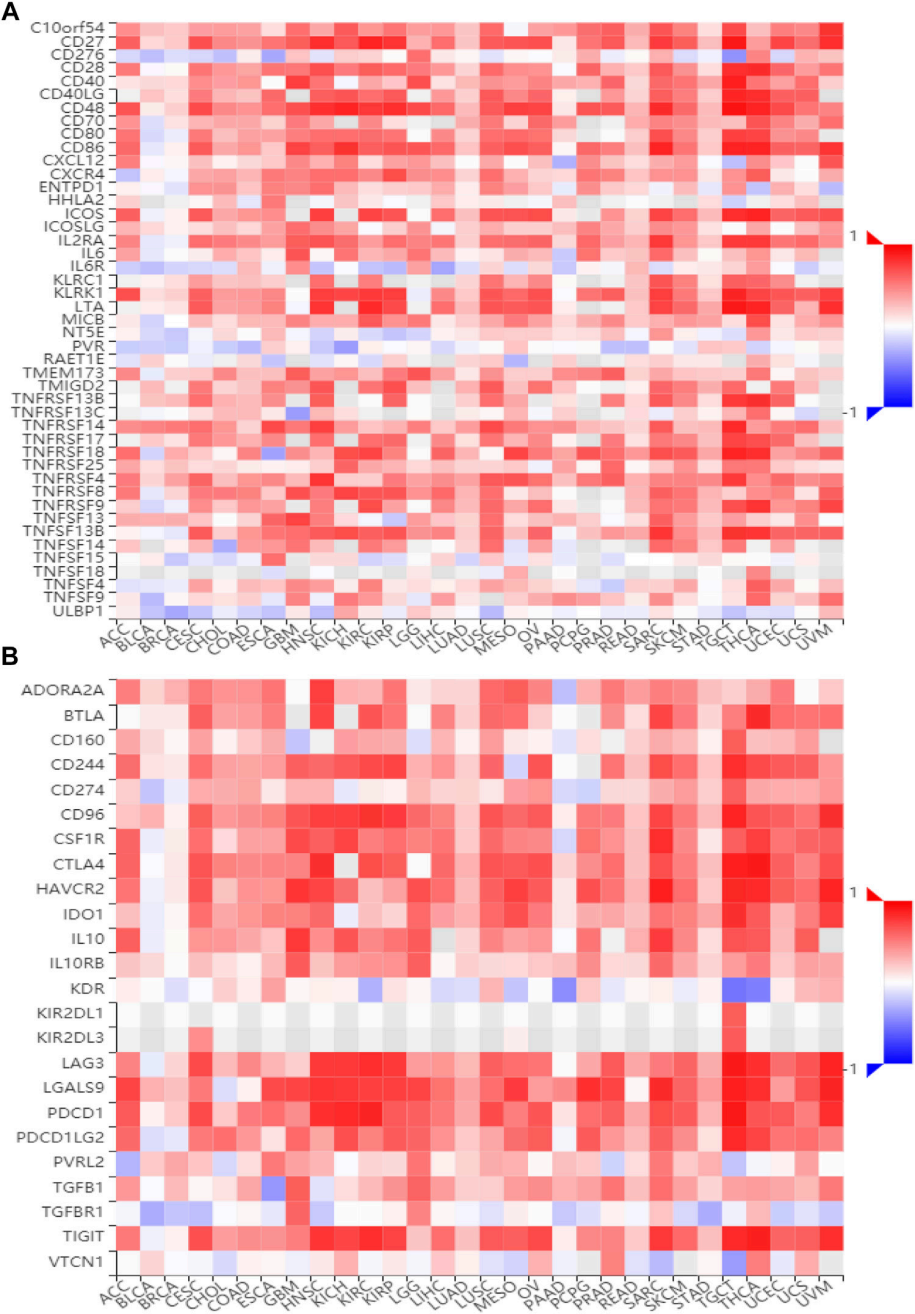
Correlation between BATF and immune regulator expression. **(A)** The expression correlation between BATF and immune stimulators. **(B)** The expression correlation between BATF and immune inhibitors.

**FIGURE 8:**
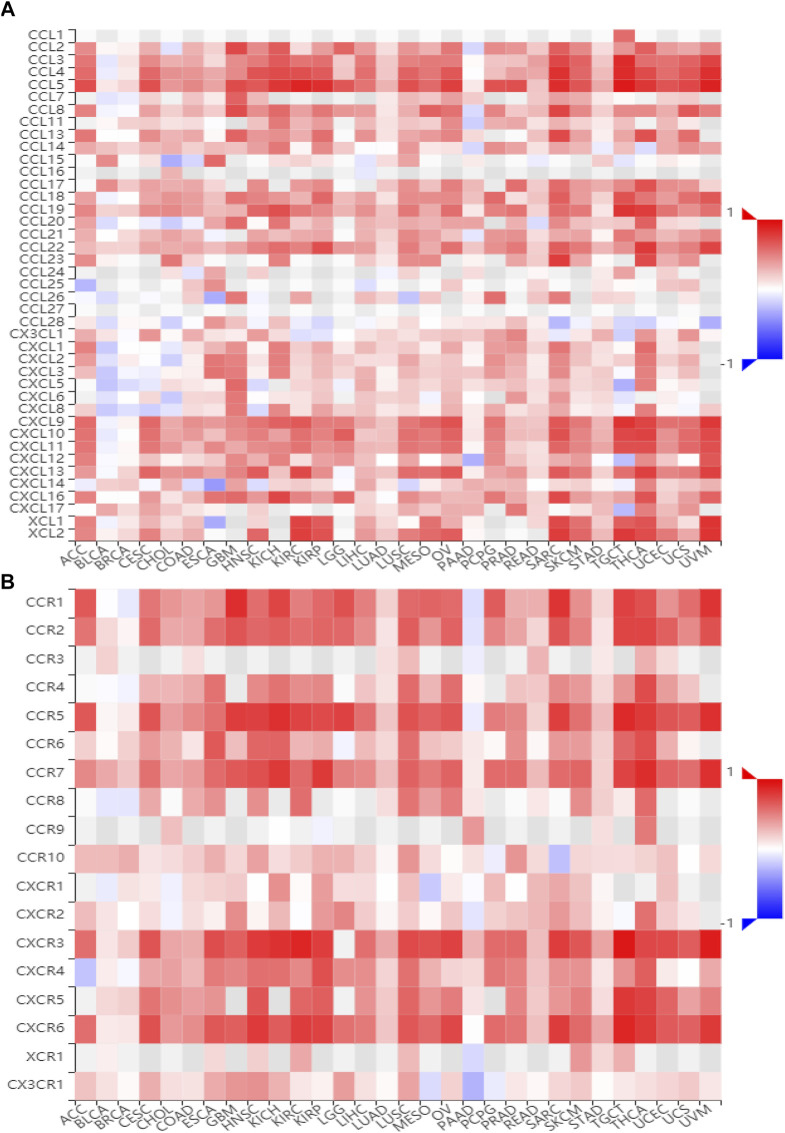
Correlation between BATF and chemokine and related receptor expression. **(A)** The expression correlation between BATF and chemokines. **(B)** The expression correlation between BATF and chemokine receptors.

To gain insights into the potential mechanisms of BATF in cancers, we performed KEGG analysis. As shown in Supplementary Figure S4, BATF was positively correlated with the natural killer cell–mediated cytotoxicity, B-cell receptor signaling pathway, programmed death ligand one expression and the programmed death 1 (PD-1) checkpoint pathway in cancer, antigen processing and presentation, T-cell receptor signaling pathway, cytokine–cytokine receptor interaction, Th1 and Th2 cell differentiation, and cell adhesion. To validate these findings, we also explored correlations between BATF expression and immunomodulators and chemokines. As shown in Figures 7, 8, BATF was positively associated with most immunostimulators, immunoinhibitors, chemokines, and related receptors. In addition, BATF expression was also highly correlated with almost all MHC molecule genes (Supplementary Figure S5A). These results indicate that BATF might regulate immune cell infiltration *via* an immune-related pathway.

### BATF Interacts With BATF3 and IRF4

**FIGURE 9:**
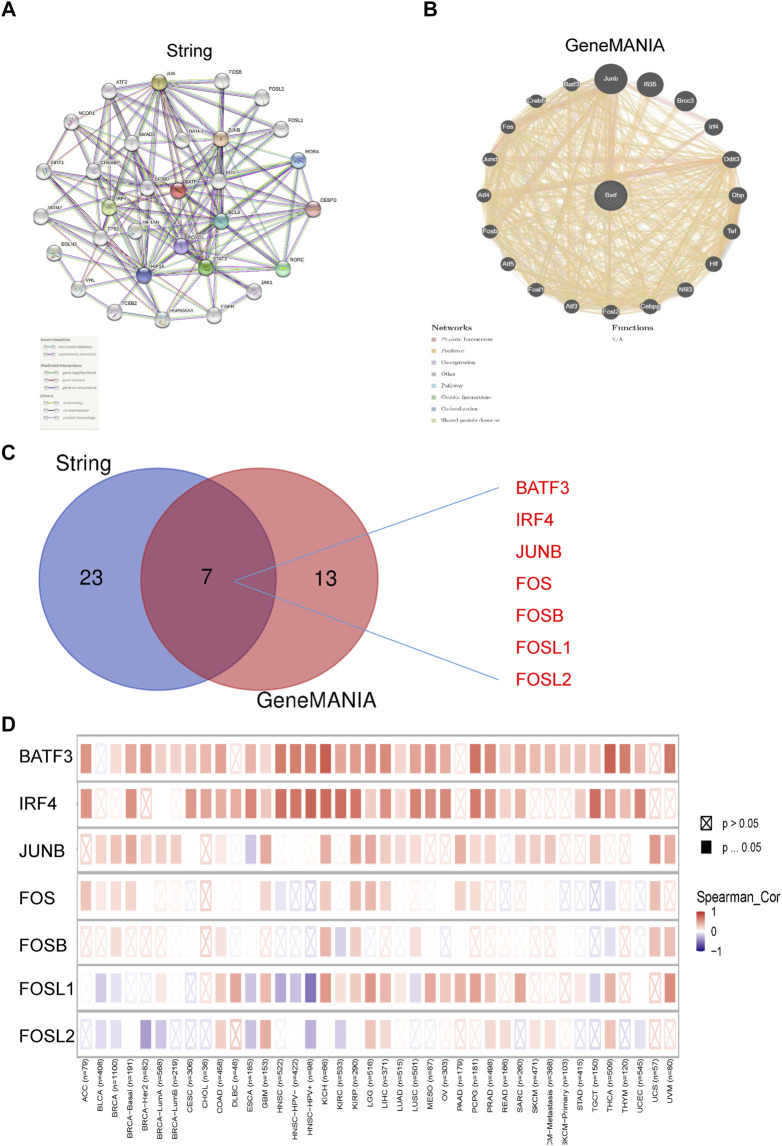
PPI regulatory network of BATF. **(A)** PPI regulatory network of BATF derived from String tool. **(B)** PPI regulatory network of BATF derived from GeneMANIA tool. **(C)** An intersection analysis of BATF-related genes between String derived PPI and GeneMANIA derived PPI network. **(D)** Correlation between BATF and BATF-related gene expression.

We have previously contended that BATF is a transcriptional factor, and we then questioned how BATF performs its roles in tumors. The STRING tool was applied to draw a protein–protein interaction network for BATF, setting a minimum required interaction score [“highest confidence (0.900)”] for obtaining BATF-binding proteins (Figure 9A). We also obtained an interactive functional association network for BATF *via* the GeneMANIA tool (Figure 9B). There were seven genes (BATF3, IRF4, JUNB, FOS, FOSB, FOSL1, and FOSL2) that overlapped (Figure 9C). Among these genes, only BATF3 and IRF4 were highly co-expressed with BATF in the majority of cancers, which suggested that BATF might carry out its role by cooperating with BATF3 and IRF4 (Figure 9D).

### Correlation Between BATF Expression and Immunotherapeutic Response

After knowing the roles of BATF in immune infiltration and immune checkpoint pathway in cancer, we wondered whether there is a correlation between BATF expression and immunotherapeutic response. As shown in Supplementary Figure S5B, BATF expression was highly associated with most immune checkpoint molecules in the majority of tumors. A low correlation between BATF and immune checkpoint molecules was observed in PAAD, LAML, and DLBC. Tumor mutation burden (TMB) and microsatellite instability (MSI) are vital immune-response biomarkers (Choucair et al., 2020; Kok et al., 2019). We found that BATF was positively associated with TMB in ESCA, CESC, UCS, PAAD, SARC, LGG, COAD, UCEC, OV, KIRC, and DLBC, but was negatively correlated with other tumors (Supplementary Figure S6A). In addition, a positive association between BATF expression and MSI was found in COAD, DLBC, READ, SKCM, BRCA, UVM, ESCA, PAAD, LAML, LUAD, LIHC, and KIRC as well as a negative association in other tumors (Supplementary Figure S6B). In five independent clinical cohorts, patients with melanoma, urothelial cancer, or clear cell renal cell carcinoma received anti–PD-1 therapy (Hugo et al., 2016; Riaz et al., 2017; Snyder et al., 2017; Mariathasan et al., 2018; Miao et al., 2018). Nevertheless, we did not found there is evident difference in BATF expression between the responder and non-responder groups (Table 2).

**TABLE 2:** BATF expression difference between responders and non 32 responders in various data sets.

No	PMID	Cancer type	Group	Drug	Responder	Non-Responder	Log2 (fold change)	*p* value
1	26997480	Melanoma	All	Anti-PD-1 (pembrolizumab and nivolumab)	14	12	−0.215	0.695
			MAPKi	Anti-PD-1 (pembrolizumab and nivolumab)	6	5	0.735	0.665
			Non-MAPKi	Anti-PD-1 (pembrolizumab and nivolumab)	8	7	−0.903	0.461
2	28552987	Urothelial cancer	All	Anti-PD-L1 (atezolizumab)	9	16	0.648	0.213
			Smoking	Anti-PD-L1 (atezolizumab)	5	9	0.192	0.872
			Non-smoking	Anti-PD-L1 (atezolizumab)	4	7	1.213	0.329
3	29033130	Melanoma	All	Anti-PD-1 (nivolumab)	26	23	0.609	0.276
			NIV3-PROG	Anti-PD-1 (nivolumab)	15	11	0.845	0.355
			NIV3-NAIVE	Anti-PD-1 (nivolumab)	11	12	0.403	0.673
4	29301960	Clear cell renal cell carcinoma	All	Anti-PD-1 (nivolumab)	4	8	2.049	0.114
			VEGFRi	Anti-PD-1 (nivolumab)	2	0	0	1
			Non-VEGFRi	Anti-PD-1 (nivolumab)	2	8	1.363	0.503
5	29443960	Urothelial cancer	All	Anti-PD-L1 (atezolizumab)	68	230	−0.065	0.64

### Correlation Between BATF Expression and the Sensitivity of Drugs Approved by the United States Food and Drug Administration

**FIGURE 10:**
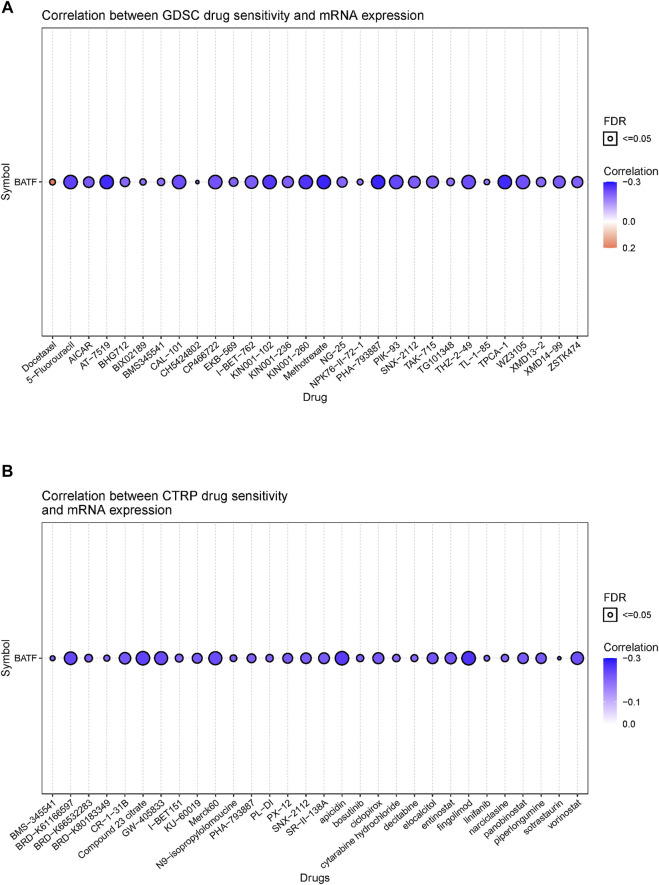
Correlation between FDA-approved drug sensitivity and BATF expression. **(A)** Correlation between GDSC drug sensitivity and BATF expression. **(B)** Correlation between CTRP drug sensitivity and BATF expression.

Although immunotherapy is a promising option for tumor treatment, chemotherapy still occupies an important position. Patients with tumors may benefit from targeting BATF with chemotherapy drugs. Hence, we investigated the correlation between BATF expression and chemotherapy sensitivity. As shown in Figure 10, there was a negative correlation between CTRP or GDSC drug sensitivity and BATF expression. In other words, patients with higher BATF expression show a lower chemotherapy response. The high expression of BATF may partially account for drug resistance in tumor chemotherapy.

### Knockdown of BATF Decreases Tumor Cell Viability

**FIGURE 11:**
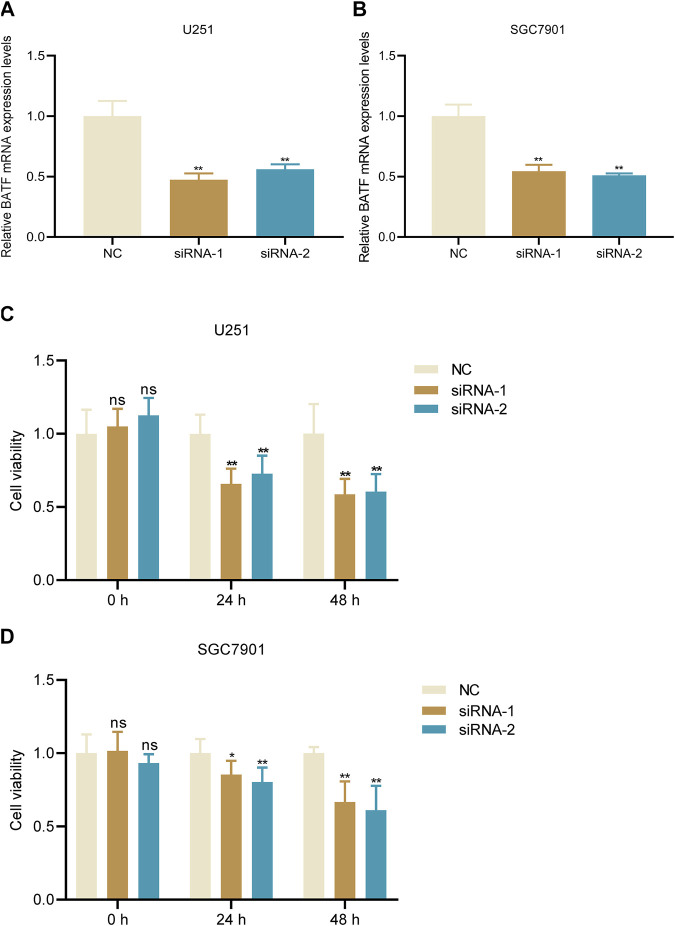
Knockdown of BATF suppresses tumor cell viability. **(A,B)** BATF knockdown decreases BATF mRNA expression in U251 and SGC7901 cells. **p < 0.01 vs. NC. **(C,D)** Knockdown of BATF inhibits U251 and SGC7901 cell viability. *p < 0.05; **p < 0.01 vs. NC.

We chose two malignant cancers glioma and gastric cancer for further analysis and experiments. Firstly, we also performed a GO and KEGG analysis of BATF in these two types of tumors. We found that BATF was not only associated with immune-cell infiltration, but also associated with cell activation in both gliomas and gastric cancer (Supplementary Figures S7, S8). Previous studies reported that BATF was crucial for T cell proliferation and exhaustion; we wondered whether BATF could also regulate tumor cell viability. Glioma U251 and gastric cancer SGC7901 cell lines were used for *in vitro* assay. As shown in Figures 11A,B, tumor cells transfected with BATF siRNA significantly downregulated BATF mRNA expression. We found that both U251 and SGC7901 cell viability were attenuated following BATF knockdown (Figures 11C,D). These results indicated that BATF not only regulated immune-cell infiltration, but also modulated tumor cell proliferation.

## Discussion

BATF was initially identified in specific human tissues and functioned as a modulator of the AP-1 transcription complex in 1995 (Dorsey et al., 1995). Subsequent research found that BATF was mainly distributed in hematopoietic cells. Varying expression levels of BATF might participate in the development of thymocytes (Sopel et al., 2016). In the present study, we first explored the expression profiles of BATF in 33 tumor types and found that BATF was aberrantly upregulated in the majority of tumors. In anaplastic large-cell lymphoma and non–small-cell lung cancer, elevated BATF expression was also reported, consistent with our study (Schleussner et al., 2018; Feng et al., 2020). Interestingly, however, decreased BATF expression was not observed in any tumors. DNA methylation is the most common way to regulate gene transcription. Except for PRAD, tumor samples with enforced BATF expression presented with lower methylation levels compared to those measured in corresponding normal tissues. Although BATF was upregulated in PRAD samples, methylation levels of PRAD are higher than those in normal tissues. We speculated that BATF expression in PRAD might be regulated by transcriptional modifications like m6A modification. However, no such study to date has reported the correlation between m6A and BATF in PRAD. In spite of the fact that BATF expression was increased in most tumors, BATF was only associated with clinicopathological stages in BLCA and KIRC, which indicated that BATF is steady during tumor progression into higher grades in other tumors. As for whether BATF has roles in BLCA and KIRC progression, further exploration is required.

Previous studies have reported that BATF is significantly related with prognosis in KIRC, COAD, LAML, and UCEC (Hu et al., 2020; Cheng et al., 2021; Huang et al., 2021; Zhang and Xiao, 2020). In line with former findings, we also found that BATF is a high-risk independent prognostic factor in ACC, KIRC, LGG, LAML, and UVM. What should be noted was that, although BATF was upregulated in BLCA, CESC, HNSC, BRCA, and UCEC, patients with higher expression levels of BATF exhibit longer survival time. Our results indicated that genetic alterations might not be crucial in the prognostic roles of BATF in cancers. First, gene-alteration rates across 33 tumor types were low, i.e., no more than 3% in each tumor. Second, gene alterations had no evident effects on OS, disease free survival (DFS), disease specific survival (DSS) or Progression free survival (PFS). However, we did find that methylation levels could predicate overall survival in patients with tumors in a tissue-dependent manner.

The roles of BATF in the differentiation of and functions in leukocytes have been well explored. For example, in the absence of BATF, Th2, and Th17 responses were impaired (Schraml et al., 2009). In CD8+ T-cells, BATF cooperated with IRF4 to regulate T-cell functions and prevent T-cell exhaustion (Seo et al., 2021). Unlike those in immune cells, the roles of BATF have only been illustrated in limited tumor types. It has been reported that AP-1–BATF module is vital for anaplastic large-cell lymphoma growth, and survival (Schleussner et al., 2018). In non–small-cell lung cancer, BATF knockdown suppressed tumor cell proliferation, while it promoted cell apoptosis (Feng et al., 2020). Our present results also indicated that BATF is important for tumor cell viability. Besides, the results of GO analysis indicated that BATF was dominantly involved in leukocyte cell–cell adhesion, leukocyte proliferation, and leukocyte activation. Related signaling pathways include cytokine–cytokine receptor interaction, PD-L1 expression and the PD-1 checkpoint pathway in cancer, Th1 and Th2 cell differentiation, antigen processing and presentation, T-cell receptor signaling pathway, natural killer cell–mediated cytotoxicity, and cell-adhesion molecules. Moreover, we also found that BATF might carry out its roles *via* cooperating with BATF3 and IRF4. All these findings indicate that BATF acts as a vital transcriptional factor in regulating tumor immune infiltration *via* an immune-related pathway. In addition, our present study found that BATF and IRF4 were co-expressed in tumors, which suggests that BATF may play its roles in tumors and immune cells in a similar way (Seo et al., 2021).

After discerning the potential roles of BATF in tumors, we wondered whether BATF expression could predict chemotherapy and immunotherapy response in tumors. We found that there is a negative correlation between BATF expression and amounts of FDA-approved drug sensitivity, which indicated that patients with higher BATF expression had lower chemotherapy sensitivity. Our present study showed that BATF expression was highly correlated with immune checkpoints in amounts of tumors. Considering the fact that BATF plays vital roles in CD8+ T-cells, we speculated that there are two strategies for tumor immunotherapy by focusing on BATF. First, the exhaustion of CD8+ cells is a common cause of the failure of immunotherapy, and the cooperation of BATF with IRF4 could counter this exhaustion in tumor-infiltrating CAR T-cells (Sopel et al., 2016). In other words, if CAR T-cells are steadily expressing BATF, progress in tumor immunotherapy will occur. Second, the exhaustion of CD8+ cells expressed excessive levels of inhibitory receptors like PD-1, LAG3, and CTLA-4, which were sensitive to an immune checkpoint blockade (Park et al., 2019; Jiang et al., 2020). For patients with high BATF expression levels, a combination of immune checkpoint blockades and anti-BATF therapy may improve survival times.

In summary, our present study found that BATF was aberrantly expressed in various tumors. Besides, BATF expression was associated with overall survival of patients with tumors. The abnormal expression of BATF participated in regulating immune-cell infiltration and predicted immunotherapy response in tumors. Our present findings may provide new insights into tumor immunotherapy.

## Data Availability

The original contributions presented in the study are included in the article/Supplementary Material, further inquiries can be directed to the corresponding author.

## Glossary

BATF: Basic leucine zipper activating transcription factor–like transcription factor
ATF: Activating transcription factor
CTLs: Cytotoxic CD8+ T cells
GO: Gene Ontology
KEGG: Kyoto Encyclopedia of Genes and Genomes
CAR: Chimeric antigen receptor
GETx: Genotype-Tissue Expression
CNA: Copy number alteration
TCGA: The Cancer Genome Atlas
GDC: Genomic Data Commons
TMB: Tumor mutation burden
MSI: Microsatellite instability
FDA: United States Food and Drug Administration
PD-1: Programmed Death 1
OS: Overall Survival
PFS: Progression Free Survival
DFS: Disease Free Survival
DSS: Disease Specific Survival
PPI: Protein-Protein Interaction
ACC: Adrenocortical carcinoma
BLCA: Bladder Urothelial Carcinoma
BRCA: Breast invasive carcinoma
CESC: Cervical squamous cell carcinoma and endocervical adenocarcinoma
CHOL: Cholangio carcinoma
COAD: Colon adenocarcinoma
DLBC: Lymphoid Neoplasm Diffuse Large B-cell Lymphoma
ESCA: Esophageal carcinoma
GBM: Glioblastoma multiforme
HNSC: Head and Neck squamous cell carcinoma
KICH: Kidney Chromophobe
KIRC: Kidney renal clear cell carcinoma
KIRP: Kidney renal papillary cell carcinoma
LAML: Acute Myeloid Leukemia
LGG: Brain Lower Grade Glioma
LIHC: Liver hepatocellular carcinoma
LUAD: Lung adenocarcinoma
LUSC: Lung squamous cell carcinoma
MESO: Mesothelioma
OV: Ovarian serous cystadenocarcinoma
PAAD: Pancreatic adenocarcinoma
PCPG: Pheochromocytoma and Paraganglioma
PRAD: Prostate adenocarcinoma
READ: Rectum adenocarcinoma
SARC: Sarcoma
SKCM: Skin Cutaneous Melanoma
STAD: Stomach adenocarcinoma
TGCT: Testicular Germ Cell Tumors
THCA: Thyroid carcinoma
THYM: Thymoma
UCEC: Uterine Corpus Endometrial Carcinoma
UCS: Uterine Carcinosarcoma
UVM: Uveal Melanoma
