# Supplementary material for: Evidence of Omics, Immune Infiltration, and Pharmacogenomics for BATF in a Pan-Cancer Cohort
Source: Front Mol Biosci. 2022 Apr 29;9:844721. doi: 10.3389/fmolb.2022.844721 (PMC9098817; doi:10.3389/fmolb.2022.844721)
Supplement: Supplementary file 1 [file DataSheet1.docx]

**Figure Legends:**

**
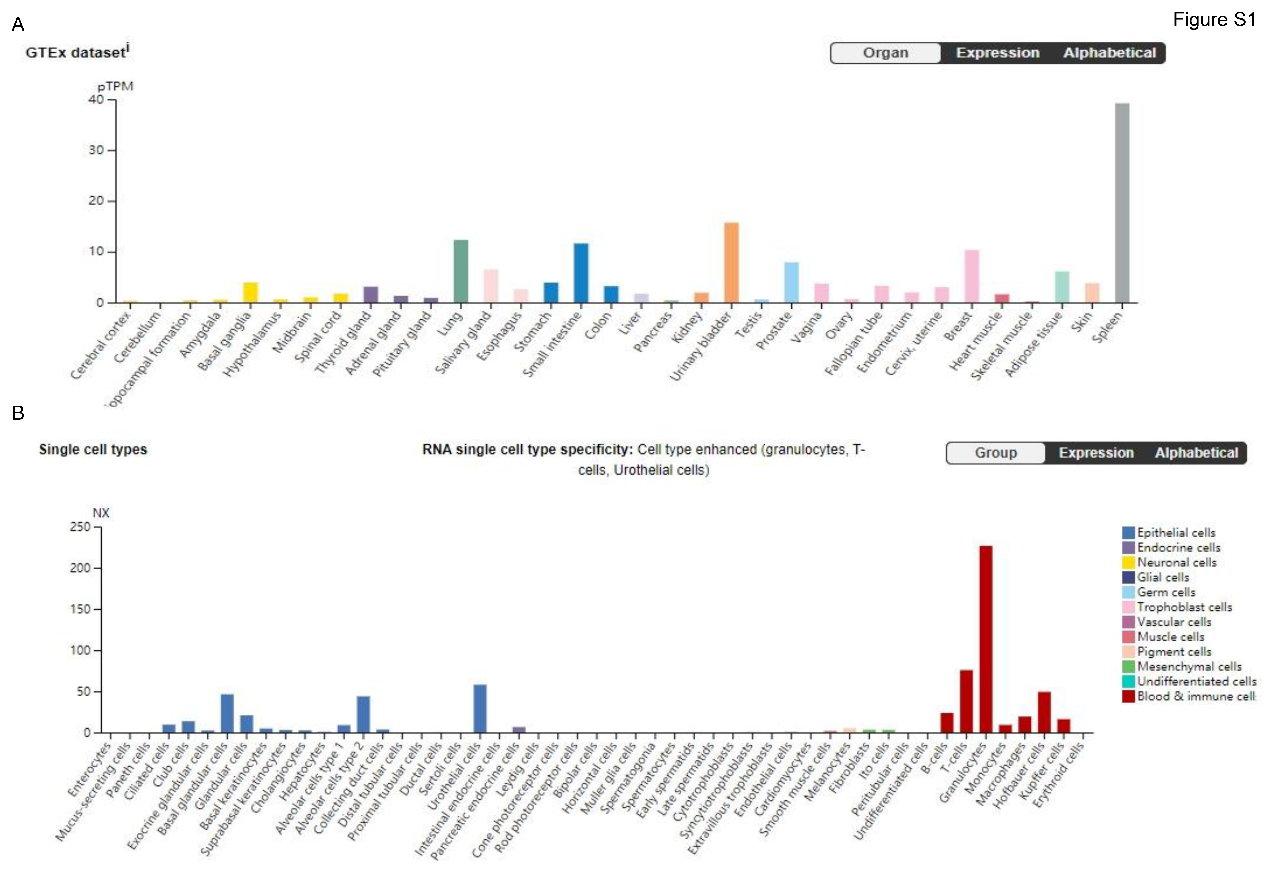
**

**Figure S1 Expression of BATF in normal tissues and single cell types.** A. BATF expression levels in normal tissues B. BATF expression levels in single cell types.


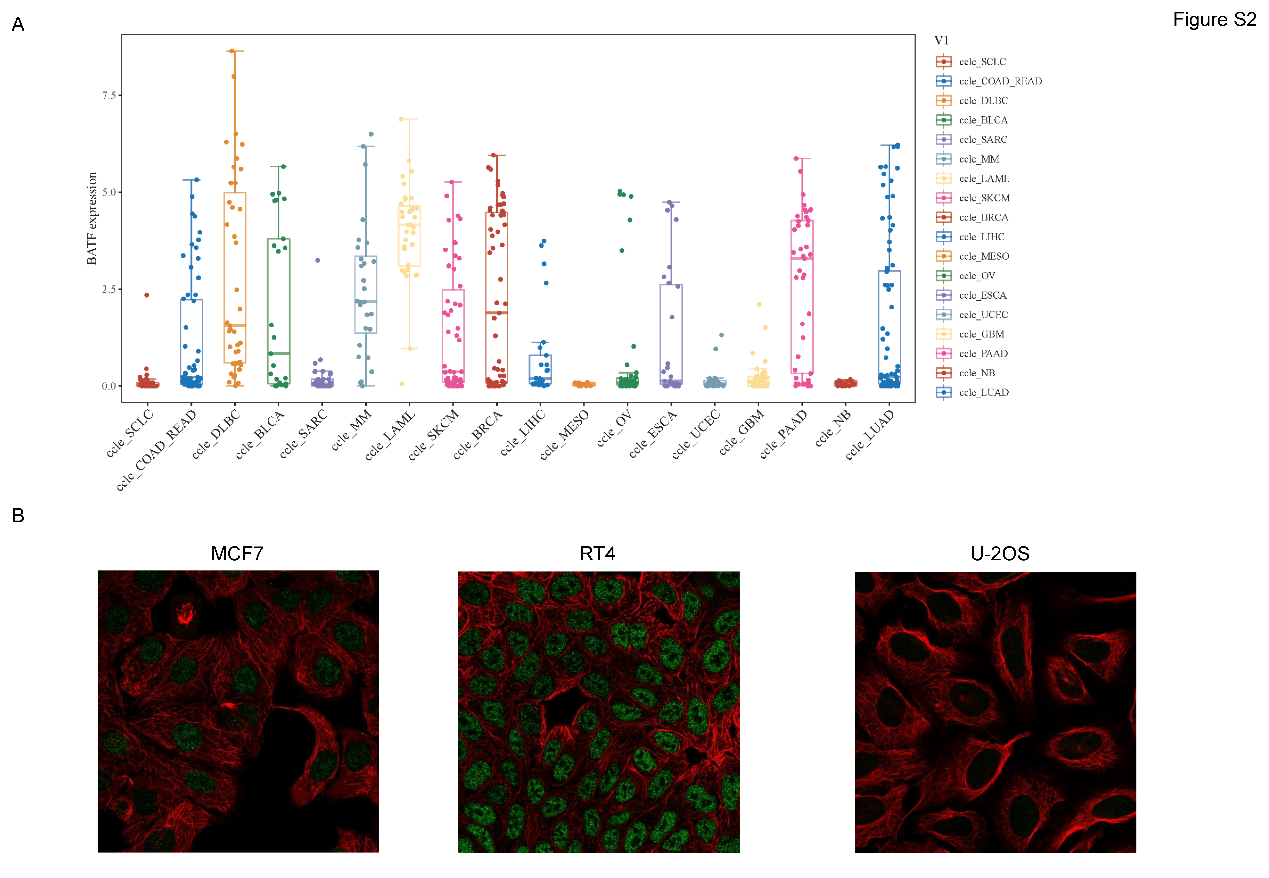


**Figure S2 Expression and distribution of BATF in tumor cell lines.** A. Expression of BATF in tumor cell lines. B. Distribution of BATF protein in tumor cell lines.


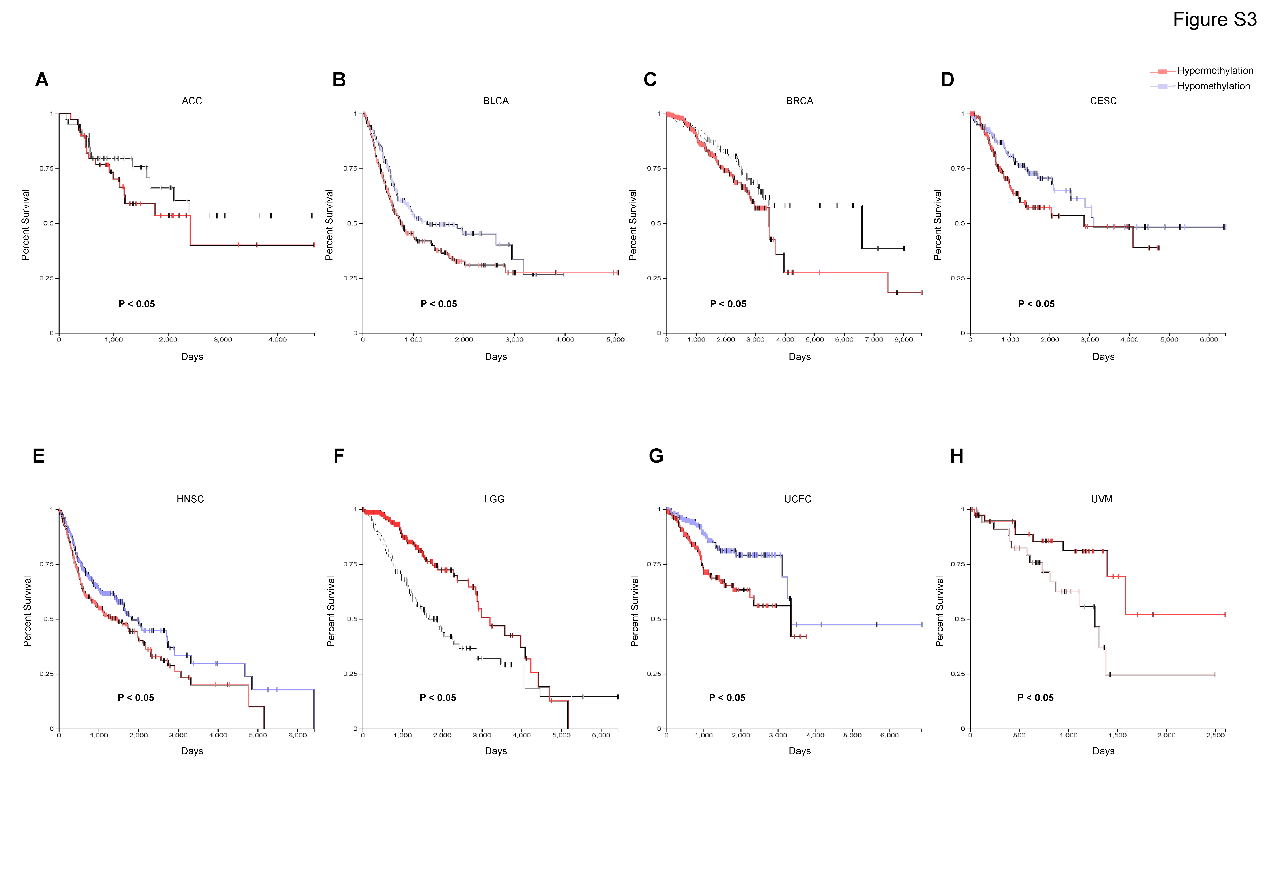


**Figure S3 Effects of BATF methylation levels on overall survival.**

**
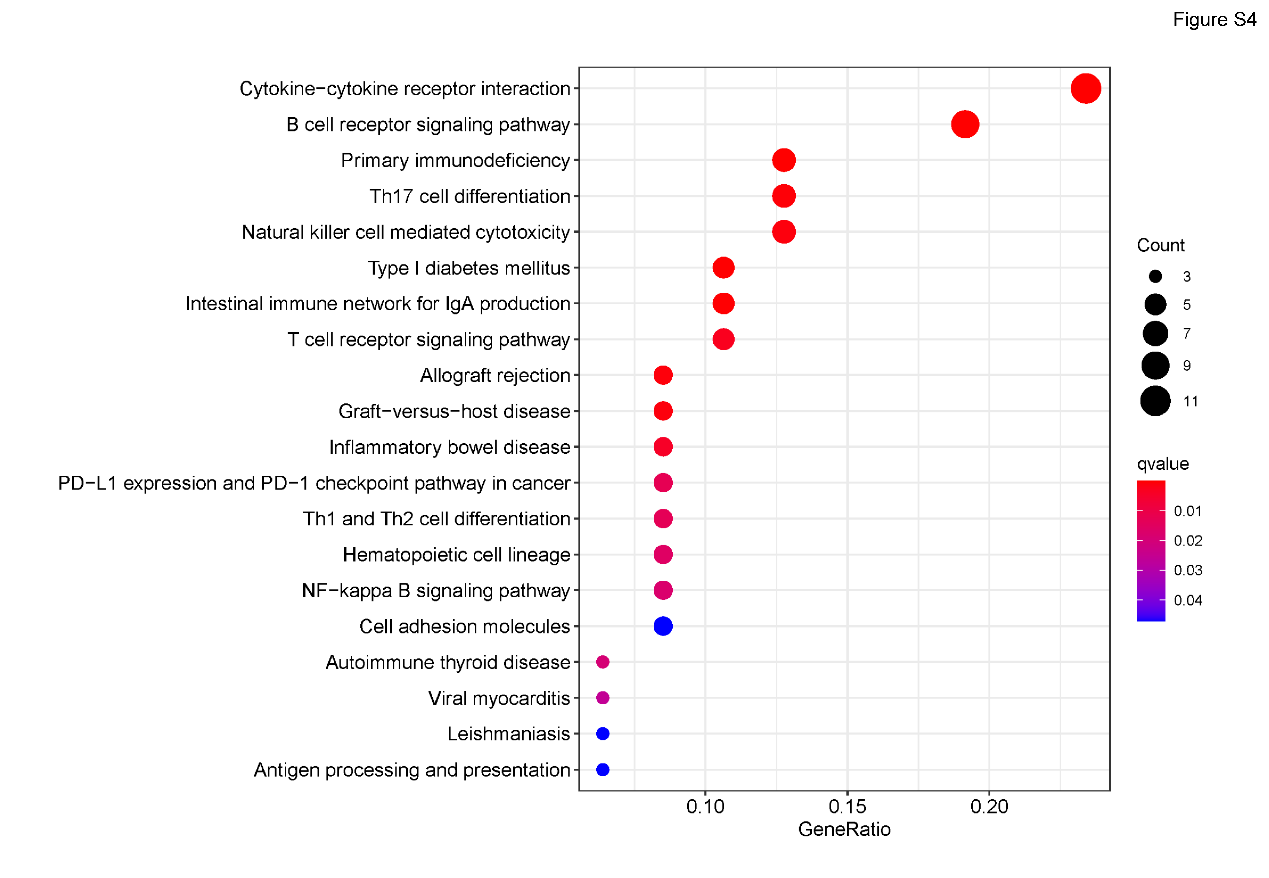
**

**Figure S4 KEGG pathway analysis of BATF.**

**
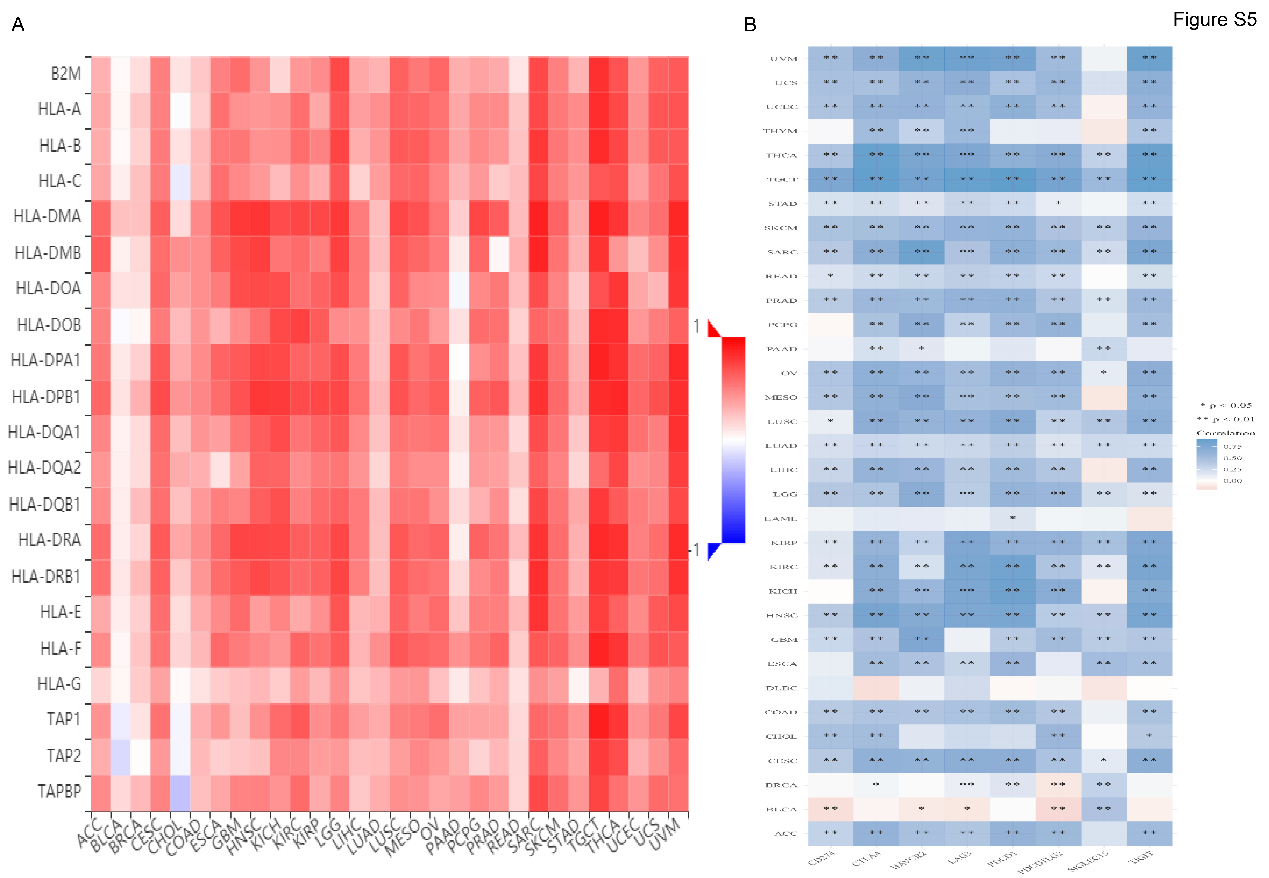
**

**Figure S5 The expression Correlation between BATF and MHC molecules and immune checkpoints.** The expression correlation between BATF and MHC molecules. B. The expression correlation between BATF and immune checkpoints.


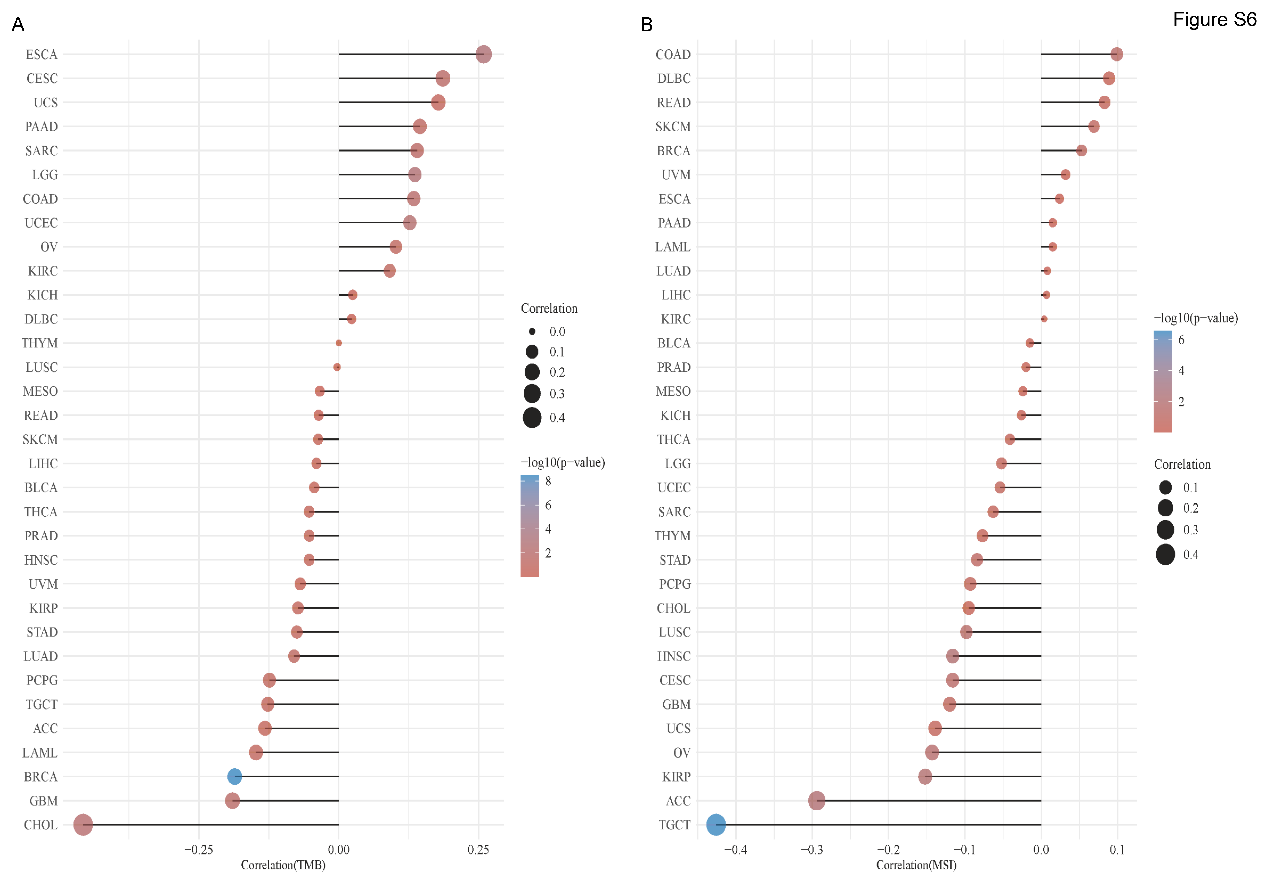


**Figure S6 Correlation between BATF expression and TMB and MSI score.** A. Correlation between BATF expression and TMB score. B. Correlation between BATF expression and MSI score.


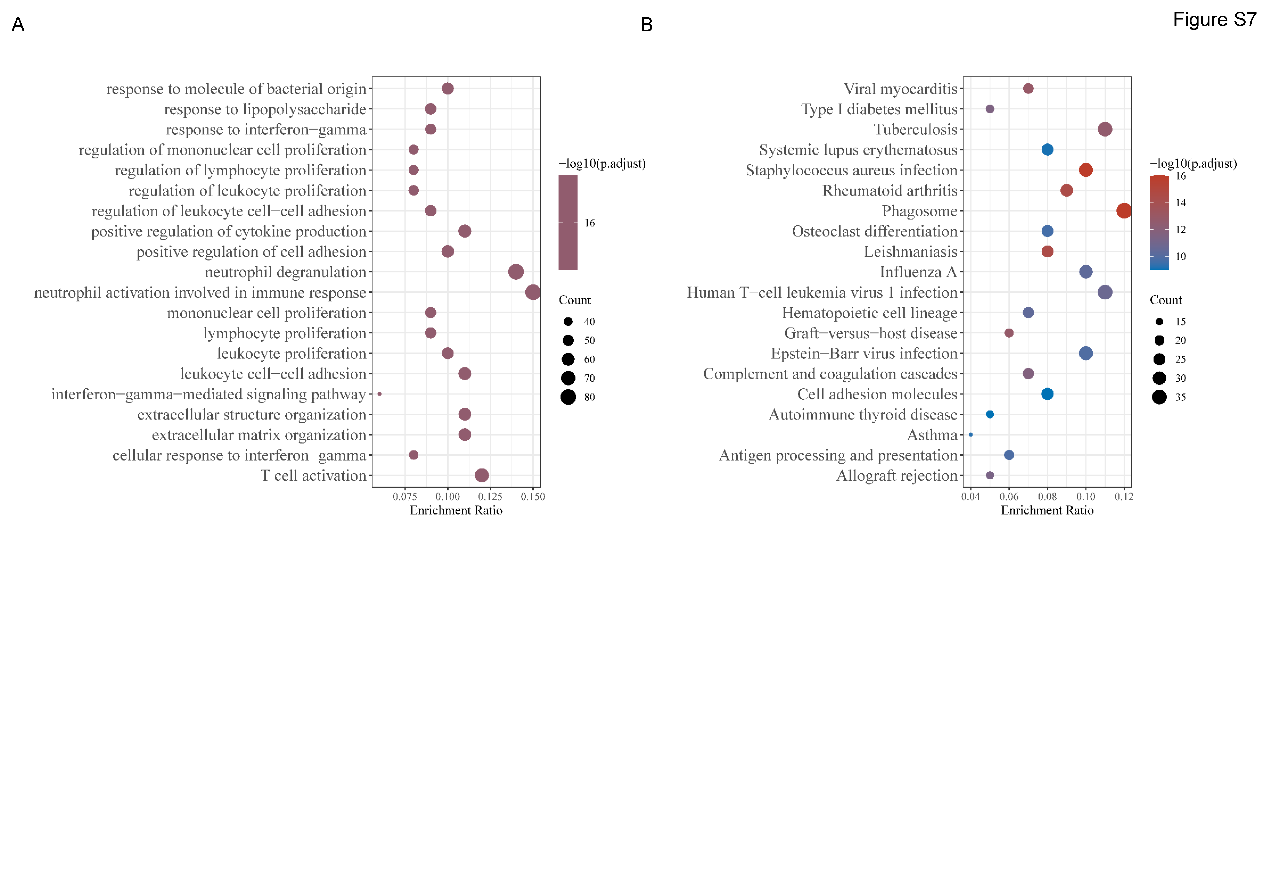


**Figure S7 GO and KEGG pathway analysis of BATF in gliomas.** A. GO analysis of BATF in gliomas. B. KEGG pathway analysis of BATF in gliomas.


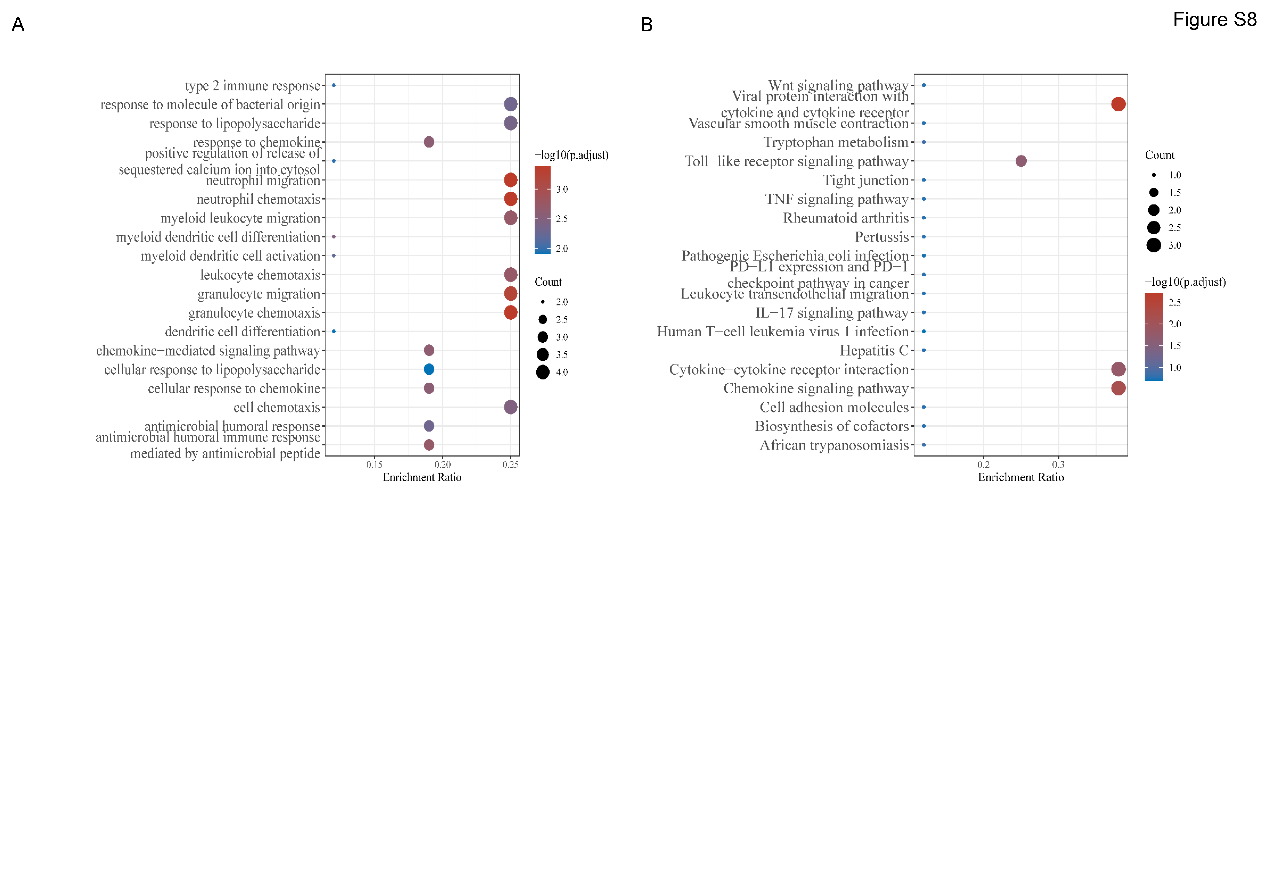


**Figure S8 GO and KEGG pathway analysis of BATF in gastric cancer.** A. GO analysis of BATF in gastric cancer. B. KEGG pathway analysis of BATF in gastric cancer.
